# Determining the Distributions of Components inside Metal–Organic Framework Thin Films with an Ar-Gas Cluster Ion Beam (Ar_1000,2500_^+^) and Ar^+^ Cosputter via Secondary Ion Mass Spectrometry

**DOI:** 10.1021/acsami.5c05778

**Published:** 2025-06-17

**Authors:** Peng-Hsuan Chiang, Pochun Hsieh, Cheng-Hung Hou, Yun-Wen You, Man-Ying Wang, Ting-Jia Yang, Jing-Jong Shyue

**Affiliations:** † Research Center for Applied Sciences, 38017 Academia Sinica, Taipei 11529, Taiwan; ‡ Department of Materials Science and Engineering, National Taiwan University, Taipei 10617, Taiwan; § Program in Semiconductor Devices, Materials, and Hetero-integration, Graduate School of Advanced Technology, National Taiwan University, Taipei 10617, Taiwan

**Keywords:** Time-of-Flight Secondary Ion Mass Spectrometry (ToF-SIMS), Gas Cluster Ion Beam (GCIB), Depth Profile, Cosputter, Metal−Organic Framework (MOF)

## Abstract

Metal–organic frameworks (MOFs) are widely used as functional porous materials because of their high specific surface area, adjustable pore size, and functional groups in their structure. Understanding the spatial distribution of guest molecules inside MOFs may help further advance the development of MOFs and provide more insights into their application in various fields. However, analytical techniques that can directly obtain the distribution of organic guests inside MOF materials are scarce. In this work, the UiO-66 MOF was used as a model MOF to validate the experimental parameters for constructing an authentic depth profile with a time-of-flight secondary ion mass spectrometer (ToF-SIMS). In the analysis phase, pulsed C_60_^+^ was used as the primary ion beam to generate molecular secondary ions. In the sputter phase, sets of Ar gas cluster ion beams (Ar-GCIB, Ar_n_^+^) with different energy densities (energy per atom, E/n = 2–20 eV/atom) and atomic Ar^+^ with different kinetic energies and current densities were used to cosputter the samples. The results show that when only Ar-GCIB is used, the sputtered ions cause less damage to the sample and preserve the chemical structure of the organic components as the E/n decreases. However, preferential sputtering occurs because the removal rate of inorganic nodes is much lower than that of the organic linkers of MOFs. Eventually, the inorganic components remaining on the surface prevent subsequent analysis. When cosputtered with Ar^+^, the auxiliary atomic ions increase the sputter rate of the inorganic node, eliminate damage to the chemical structure, and alleviate the preferential sputtering between organic and inorganic components. Higher voltages and higher current densities (500 V, 5 × 10^–6^ A/cm^2^) of Ar^+^ yielded the most realistic results. In summary, to obtain a realistic component distribution inside the MOF, the use of Ar-GCIB—Ar^+^ cosputtering is necessary. Based on low energy density (E/n = 4 eV/atom) of Ar-GCIB and optimized Ar^+^ cosputter, the distributions of inorganic nodes, organic linkers, and guest molecules inside the MOF films were reliably and thoroughly identified. This work presents a generalizable direct method for determining the distribution of molecules within MOF composites.

## Graphical abstract

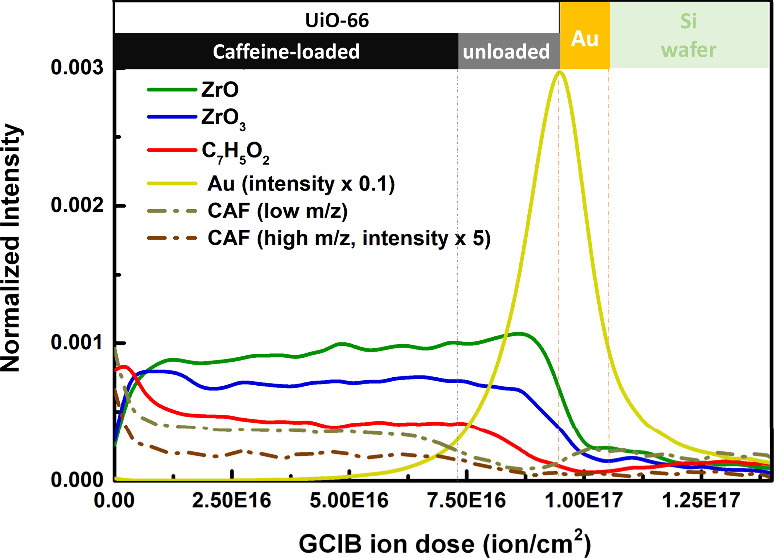

## Introduction

Metal–organic frameworks (MOFs), also known as porous coordination polymers (PCPs), have been demonstrated to be highly promising functional materials for various applications, including drug delivery,^1–4^ biomedical imaging,^1,5^ solid–phase extraction,^6–9^ electrochemical/photochemical catalysis,^10–13^ gas separation,^14^ etc. According to the definition given by Yaghi,^15^ an MOF is a solid material composed of inorganic and organic building units, i.e., metal or metal oxide nodes and molecular linkers. In a *Science* review article,^14^ some of the common building blocks are summarized. The functionalities of MOF and its selectivity toward the adsorption of molecules originate mostly from its extremely high porosity and the chemically tunable affinity of cavity walls. To achieve flexible chemical affinity of the cavity wall, postsynthetic modification (PSM) can also be applied to replace nodes and/or linkers.^16^ It has also been demonstrated that both the porosity and surface affinity can be effectively modified by varying the length of linkers^17^ and introducing the desired functional groups on the linker,^18^ respectively. As a result, molecular compounds of different sizes, e.g., vitamin-B_12_, myoglobin and green fluorescent protein, can be selectively loaded into MOF cavities,^17^ and the adsorption behavior of gas molecules, e.g., H_2_ and CO_2_, can be tailored.^18^

Well-controlled capture and release of the adsorbate is critical to the application of MOFs.^19^ In general, by engineering the cavity size and modifying its surface with the required functional group, a wide variety of adsorbates, so-called “cargo” or “guest”, can be selectively captured and encapsulated into the cavity of the MOF. It is also possible to synthesize a MOF with the desired cargo as the linker and then release it through decomposition. Both the encapsulation and assembly strategies are able to provide extremely high loading efficiencies for guests due to the high surface area and large number of adsorption sites inside the MOF structure. Depending on the application, the guest molecule can be captured and then transferred, reacted, released, etc. To understand these processes and the transportation/diffusion of molecules inside the MOF to further advance the development of the MOF, the distribution of guests and their molecular structure (after optional chemical reactions) inside the MOF should be characterized with sufficient spatial resolution and chemical sensitivity. Unfortunately, methods that can directly obtain the distribution of diffusion-loaded organic guests or modified sites postsynthesis inside MOF materials are scarce.^20^ It is clear that analytical techniques capable of directly determining the component distribution inside MOF composites are lacking, and developing suitable techniques to support the scientific development of MOFs is crucial.

To study the diffusion of guests inside a MOF, indirect methods are generally used. For example, when ibuprofen and aspirin are used as modeling molecules, their diffusion into and out of MIL-100­(Fe), MIL-127­(Fe) and UiO-66 particles dispersed in water are studied.^21^ However, instead of directly measuring the distribution of guests inside the MOF, high-performance liquid chromatography (HPLC) is used to determine the concentration of guest molecules in the solution, and the change in concentration at given times is used to refer to the amount of guest loading or unloading. To determine the distribution of guests or modified sites inside a MOF, a more direct analytical technique is desired. Owing to its high spatial resolution (<100 nm lateral and ∼ nm deep) and limit of detection (ppm level), secondary ion mass spectrometry (SIMS) is among the most important analytical tools for determining the dopant distribution inside solids.^22^ However, SIMS was underused to study organic–inorganic composite materials such as MOFs because high-energy ion bombardment can easily break chemical bonds and destroy organic components. In a review article on the use of ion beams to analyze MOFs,^23^ the destructive nature of SIMS was emphasized; hence, other methods, including medium-energy ion scattering (MEIS) and elastic recoil detection analysis (ERDA), were the focus. Although SIMS is indeed destructive, by restricting the ion fluence within the static regime (<1 × 10^12^ ions·cm^–2^) to limit the total amount of damage to chemical structures, few studies have utilized time-of-flight SIMS (ToF-SIMS) to verify the location^24,25^ and linker exchange^26^ of surface-mounted MOF films. However, under the static limit, it is not possible to obtain information from the subsurface; hence, the diffusion of guest and modified sites inside the MOF cannot be determined. To investigate the early stage structural development of a MOF material, an elaborate sample preparation method utilizing a liquid SIMS sample holder was then reported.^27,28^

Currently, analytical techniques that can be generally applied to obtain the microstructure and component distribution inside MOFs have not yet been reported. Despite the sufficiently high spatial resolution of SIMS, the main challenge is the organic–inorganic hybrid nature of the MOF. To acquire a component distribution beyond the surface, a standard strategy is a sputter depth profile, where an analysis causing little or no structural alteration is applied; then, the outermost surface is sputtered away by a high fluence of ions to expose the subsurface for the next analysis. The change in the acquired signal intensity as a function of sputter time is then used to construct the depth profile. Unfortunately, for organic–inorganic composites such as MOFs, significant analysis artifacts, such as accumulations of altered molecular structures and chemical compositions on the sputtered surface, are frequently observed due to the ion cleavage of chemical bonds, preferential sputtering of some components or ion-induced mixing of some components. These artifacts are more significant for organic components when damage is introduced faster than be removed. Although these artifacts have long been overlooked, they have drawn attention recently, and few publications have addressed them for the righteous study of hybrid organic–inorganic perovskites.^29,30^ In these prior reports, various strategies, such as the use of different types of ion beams (reactive or not reactive; small atomic or large cluster), acceleration voltages, and the molecular weights of ions, are employed to find suitable experimental parameters in a trial-and-error sense before true scientific augments are made.

While MOFs are hybrid materials with organic and inorganic components, artifacts similar to those generally observed on organohalide perovskites can be expected. As a result, guest uptake is rarely verified by actual characterization of the component distribution inside the MOF, especially for molecules composed of the elements that are already present in the vacant MOF. In fact, depth profiles of guest-MOF composites have been reported using atomic ions (Ar^+^, O_2_^+^, Bi_*x*_^+^) as the sputter source. However, only elemental ions (Si^–^, ZnO^–^,^31–37^ I^–^,^20^ *etc*.) or small molecular fragments (C_4_N_2_^+^,^36^ CN^–^,^20,32^ *etc*.) are used in the depth profile for discussion. For guests composed of the same elements as organic linkers, the guest and linker signals cannot be differentiated by these elemental ions and small molecular fragments so that their spatial distributions cannot be discussed. If the guest-MOF composite contains isomers, it will be even more difficult to differentiate the signals because they have the same *m*/*z*. These unsatisfactory results also indicate that proper characterization of guests in MOFs is difficult and needs to be addressed.

After more than two decades of development in polyatomic and cluster ion sources such as SF_5_^+^,^38,39^ C_60_^+^,^40–44^ Au_*x*_^+^,^45^ Bi_*x*_^+^,^44^ Ar_1000–4000_^+^ ^46^ and (H_2_O)_n_^+^,^47^ they have enabled promising approaches to minimize the sputter damage of organic species and yield more realistic depth profiles. Compared with that of atomic ions, the significantly reduced energy density (energy/atoms, E/n) greatly limits the penetration depth of these bulky ions, and energy exchange with solids is restricted to the outermost surface. As a result, the subsurface is damaged to a lesser extent. Furthermore, a nonlinearly enhanced sputtering yield can be achieved to increase the secondary ion yield and mask sputter induced damages. All these features contribute to better preservation of secondary molecular ions from the remaining surface for subsequent analysis. As a result, in the dynamic regime of ion sputtering, parent ions (i.e., unfragmented molecular ions) can be obtained for direct chemical identification based on the molecular weight. For MOF, only a few reports have utilized C_60_^+^ ^34^ and Ar_1500_^+^ ^48^ cluster ions to perform sputter depth profiling. However, these works reported only elemental species. Therefore, the potential of applying cluster ions to obtain molecular information directly within MOFs has not yet been realized.

In this work, UiO-66 was used as a modeling MOF, and caffeine was used as the molding guest molecule. ToF-SIMS depth profiles using ion beams of energy per atom ranging from 500 to 200 V Ar^+^ (E/n = 500–200 eV/atom), 20 kV C_60_^+^ (E/n = 333 eV/atom) to 20–5 kV Ar_1000–2500_^+^ (E/n = 20–2 eV/atom) were compared. For a lower E/n, the low sputter rate prevented the profile from passing through the whole film in a reasonable amount of time. Furthermore, damage inevitably accumulated due to the low sputter rate; hence, an artifact was introduced in the depth profile. A higher E/n, which could remove surfaces more efficiently, also introduced more significant chemical damage to the molecular structure on the remaining surface; hence, it cannot provide correct results. By cosputtering surfaces with both high and low E/n ions at the same time to balance the introduction and removal of structural damage, an ideal depth profile can be obtained. Therefore, the distribution of modeling caffeine inside UiO-66 after a loading process can be determined and could be applied to gain more insight into various applications of MOFs.

## Experimental Section

### Synthesis of UiO-66

UiO-66 was synthesized according to articles from Y. Huang^49^ and K. M. Choi.^50^ In brief, 49.8 mg of 1,4-benzenebicarboxylate (ACROS Organics, Belgium) and 30 μL of triethylamine (TEA, Alfa Aesar, UK) were dissolved in 5 mL of N,N-dimethylformamide (DMF, Sigma–Aldrich, USA) to obtain stock solution 1. A total of 66.8 mg of ZrCl_4_ (ACROS Organics, Belgium) and 1.38 mL of acetic acid (SHOWA, Japan) were dissolved in 5 mL of DMF to form stock solution 2. These stock solutions were combined, sealed and heated to 85 °C for 24, 20.5, or 20 h. After the solvothermal reaction, the resulting white precipitates were collected by a centrifuge (9000 rpm, 10 min) and then sonicated in DMF three times to remove residual reactants. The product was then sonicated in methanol (ACROS Organics, Belgium) for 1 day then collected by centrifugation for three times. The final product of UiO-66 was then spin coated on the Au/Cr-coated Si substrates.

### Preparation of the UiO-66 Thin Films

A 70 nm Au/Cr-coated Si wafer was cut to a size of 2 cm × 2 cm as the substrate for UiO-66 thin film formation. The substrates were immersed in ethanol and sonicated for 10 min. After ultrasonic cleaning, the substrates were dried under N_2_ flow and treated with UV-ozone for 30 min. Well-cleaned UiO-66 particles were dispersed as suspensions in absolute ethanol with a concentration of 40 mg/mL. The UiO-66 thin films were then prepared by spin-coating the suspension on the cleaned Au substrate at 3000 rpm for 30 s. After drying at room temperature, the same spin-coating process was repeated 5 times to increase the thickness and surface coverage of UiO-66.

### Loading Caffeine into the UiO-66 Films

To load caffeine into the cavity of UiO-66, a 10 mg/mL aqueous solution of caffeine was prepared. The UiO-66 films were then immersed in the solution for 72 h, and the caffeine-loaded UiO-66 thin films were dried under a N_2_ stream.

### Characterization Methods

X-ray powder diffraction (XRD) patterns of UiO-66 particles with 24, 20.5, and 20 h reaction times were obtained via an X-ray diffractometer (Rigaku TTRAX 3, Japan) with Cu Kα radiation (λ = 1.54059 Å) with a 5 ∼ 30° diffraction angle. The morphology and cross sections of the films after 5 spin-coating cycles were assessed by scanning electron microscopy (SEM, FEI Nova200 NanoSEM, Netherland) to determine the coverage of UiO-66 and its thickness from multiple samples prepared at different times. The topographies of the films formed by UiO-66 particles with different reaction times were also characterized with an atomic force microscope (AFM, diInnova SPM, USA). The 15 μm × 15 μm AFM images (256 × 256 pixels) were obtained in tapping mode using cantilevers with a stiffness of ∼ 7.4 N/m and a frequency of ∼ 160 kHz. The root-mean-square (RMS) roughness of the multiple films prepared at different times was then determined.

### ToF-SIMS Depth Profiling

The time-of-flight secondary ion mass spectrometry (ToF-SIMS) depth profiles were acquired with a PHI TRIFT V nanoTOF (ULVAC-PHI, Japan) using a dual-beam in the sputter-and-acquisition scheme (slice-and-view). A C_60_^+^ source operated at 20 kV (IOG-C60–20, Ionoptika, UK) with 0.16 nA DC (measured by the current monitor within the ion column) and ∼ 8200 Hz repetition of a 15 ns pulse was used as the primary ion beam in the analysis phase. In favor of a higher beam current and signal intensity, the beam size in this setup in the order of μm. Each spectrum was acquired by rastering the primary beam on a 50 μm × 50 μm area with an incident angle of 42°. During the 3 min acquisition time, the total primary ion dosage was 8.9 × 10^11^ ions/cm^2^, which was lower than the static limit of 1.0 × 10^12^ ions/cm^2^. The secondary ions were accelerated by pulsed 3 kV sample bias and passed through a 2 m flight path before being counted by the detector with 128 ps time resolution. Typical mass resolution (m/Δm_fwhm_) at *m*/*z* 200 is approximately 3600. Near the end of the flight path, an Ar gas-filled collision-induced dissociation cell was retrofitted in front of a 1 m long, 14 kV linear flight tube, and *m*/*z*-selected secondary ions were deflected into the collision cell to perform ToF-ToF tandem MS. During the flight of secondary ions and sample grounded, pulsed 10 V electron and 10 V Ar^+^ ion flooding was applied for charge compensation. The reported secondary ion intensity was normalized with respect to the total ion count of each acquisition phase to compensate for the fluctuation in the primary ion current. In the sputter phase, C_60_^+^ operated at 20 kV and different acceleration voltage of Ar-GCIB (06–2100, ULVAC-PHI, Japan) were applied with and without Ar^+^ (06–350, ULVAC-PHI, Japan) cosputter. The apparatus used in this study is equipped with a standard feature that allows cosputtering of a sample using two separate ion guns. For C_60_^+^, a current density of 2.78 × 10^–7^ A/cm^2^ with a 42° incident angle was used. For Ar-GCIB, the incident angle was 50°, and acceleration voltages of 5, 10, 15, or 20 kV with cluster sizes of 1000 and 2500, which were controlled by the gas pressure, were used to obtain E/n values of 2, 4, 6, 10, 15, and 20 eV/atom. The desired current densities of Ar-GCIB with different values of E/n during the sputter phase were fine-tuned by the condenser lens. For Ar^+^ cosputtering, acceleration voltages of 200 and 500 V were achieved by decelerating a 1000 eV beam with 800 and 500 V floating columns, respectively, with an incident angle of 45°. The parameters of the Ar^+^ cosputter beam were set to low-high, high-low, and high–high beam voltage–current densities, which were 200 V-5.00 × 10^–6^ A/cm^2^, 500 V-2.22 × 10^–6^ A/cm^2^ and 500 V-5.00 × 10^–6^ A/cm^2^, respectively. Plural experiments were conducted and representative profiles were reported.

## Results and Discussion

### Preparation of the UiO-66 Particles and Films

The synthesis of UiO-66 was based on previously reported methods.^49,50^ It took approximately 19 h for the solution to become turbulent; hence, a minimal reaction time of 20 h was selected. Figure S1 shows the XRD patterns of UiO-66 obtained at 20, 20.5, and 24 h reaction times. Similar patterns at 2θ = 7.36°, 8.48°, 12.04° and 25.74° corresponding to (111), (200), (220) and (442) of UiO-66 (COD-4512072)^51^ confirmed the formation of crystalline UiO-66.

Figure S2 shows the SEM and AFM topography of UiO-66 films spin-coated on Au/Cr-coated Si substrates. The particle size clearly increased with increasing reaction time, and a rough surface with inhomogeneous particle sizes at 24 and 20.5 h was not suitable for the ToF-SIMS depth profile. Nevertheless, at 20 h, although some cracks can be observed in the top views with an RMS roughness of 39.17 nm, 981 ± 17 nm thick films can be obtained. Therefore, UiO-66 synthesized at 20 h was selected for subsequent studies.

### ToF-SIMS Analysis of UiO-66

UiO-66 is composed of metallic nodes of the Zr_6_O_4_(OH)_4_ cluster and organic linkers of terephthalic acid. To faithfully present the structure of UiO-66, fragments that could represent the inorganic node and the organic linker need to be identified with minimal overlap to avoid interference. The secondary ion mass spectrum collected with positively charged secondary ions (Figure S3a) showed two main peaks at *m*/*z* 90 and 106, which can be assigned to [Zr]^+^ and [ZrO]^+^, respectively, according to the isotope distribution around these main peaks. However, the peak at *m*/*z* 228 matched both [ZrC_7_H_6_O_3_]^+^, which is composed of both Zr and the organic ligand, and the Zr_2_O_3_ inorganic node. Although following formula cannot be easily correlated to the known chemical structure of UiO-66, other elemental combinations, such as [Zr_2_C_4_]^+^, [Zr_2_CH_4_O_2_]^+^, [ZrC_6_H_2_O_4_]^+^, [ZrC_10_H_2_O]^+^, [ZrC_3_H_6_O_6_]^+^, etc. may also correspond to the observed peak at *m*/*z* 228. Additionally, due to the natural abundance of ^13^C, the ^12^C-enriched C_60_ source contains roughly 80% ^12^C_60_, along with inevitable isotopic variants such as ^12^C_59_^13^C_1_, ^12^C_58_^13^C_2_, and others. These heavier ^12^C_60–*x*_^13^C_*x*_ isotopologues in the primary ion beam arrive at the sample slightly later than ^12^C_60_, causing asymmetric peak tails. This effect reduces confidence in the structural determination of the peak observed at *m*/*z* 228, even though the mass deviation from [Zr_2_O_3_]^+^ is only about –5 ppm when the mass is calibrated using [In]^+^ and [In_2_O]^+^. Therefore, to confirm the structure of the secondary ion at *m*/*z* 228, it was selectively deflected to a secondary path and passed through an Ar-filled collision cell to further fragment this secondary ion to analyze its structure in terms of tandem mass (MS2). The MS2 peak from *m*/*z* 228 (Figure S3b) showed four prominent peaks at *m*/*z* 212 (P-16), 122 (P-106), 106 (P-122) and 90 (P-138). These ions can be assigned to the loss of O, ZrO, ZrO_2_ and ZrO_3_ from the precursor ion (P) at *m*/*z* 228. Since there were no C- or H-related components, *m*/*z* 228 was assigned to [Zr_2_O_3_]^+^. In summary, there is no suitable peak that can represent the organic linker with positive polarity.

In negative polarity, the resulting spectrum (Figure S3c) showed three prominent peaks at *m*/*z* 106, 122 and 138, which correspond to the structures of [ZrO]^−^, [ZrO_2_]^−^ and [ZrO_3_]^−^, respectively. These peaks represented the inorganic nodes in UiO-66. In addition, the peak at *m*/*z* 121, with an exact value slightly higher than integer, suggested that this ion is a hydrogen-containing (organic) fragment and could represent the organic linker. The stable ions at *m*/*z* 121 could be [C_3_H_5_O_5_]^−^ and [C_7_H_5_O_2_]^−^. Therefore, MS2 was conducted (Figure S3d) to determine its chemical structure. The observed product ions have *m*/*z* values of 120 (P-1), 108 (P-13), 97 (P-24), 96 (P-25), 85 (P-36), 84 (P-37), 73 (P-48), 72 (P-49), 40 (P-61) and 48 (P-73), which correspond to the loss of H, CH, C_2_, C_2_H, C_3_, C_3_H, C_5_, C_4_H, C_5_H and C_6_H, respectively. According to this pattern, *m*/*z* 121 can be identified as [C_7_H_5_O_2_]^−^, which corresponds to benzoic acid; that is, a carboxylic acid group was lost from the terephthalic acid linker, and the structure can also be written as [M-CO_2_H]^−^ (M is the terephthalic acid linker). Since there is no Zr-node related species, *m*/*z* 121 can be used to represent the organic linker. In summary, when negatively charged secondary ions were used, *m*/*z* 106 and 138 ([ZrO]^−^ and [ZrO_3_]^−^, respectively) represented metallic nodes, and *m*/*z* 121 ([C_7_H_5_O_2_]^−^, [M-CO_2_H]^−^) represented the organic linker in UiO-66. For ideal spin-coated UiO-66 films, a homogeneous distribution of metallic nodes and organic linkers along the depth can be expected. Therefore, the change in the intensity of these peaks during the depth profile can be used to evaluate the degree of sputter damage; a steady ratio of peak intensities would indicate negligible damage, and the true distribution of the components would be observed.

### Depth Profile of UiO-66 with a Single Sputter Beam

Figure 1 presents depth profiles obtained with various sputter beam parameters. The intensities of the secondary ions that represent inorganic nodes ([ZrO]^−^ and [ZrO_3_]^−^) and organic linkers ([C_7_H_6_O_2_]^−^, [M-CO_2_H]^−^) selected above are normalized with respect to the total secondary ion intensity to account for variations in the primary beam. Only 20 kV Ar_1000_^+^ and 500 V Ar^+^ had sufficient sputter rates (Figure 2a, numerical values are presented in Table S1) to remove the 981 ± 17 nm thick UiO-66 film and expose the Au substrate within a few hours. However, a broad interface between UiO-66 and Au was observed, which indicated ion-induced mixing and an blurred interface. Furthermore, the rapid decrease in [C_7_H_6_O_2_]^−^ intensity makes them unsuitable for determining the distribution of organic components. For 200 V Ar^+^ and 20 kV C_60_^+^, a slight increase in Au^–^ can be noted after ∼ 6 h, which indicates significant atomic mixing, and the interface cannot be defined to determine the sputter rate. For other beam parameters with E/n ≤ 15 eV/atom, although pseudosteady state may be established, no Au^–^ signal can be identified, which indicates that the sputter rate is too low to remove UiO-66 films even with extended sputter time.

**Figure 1:**
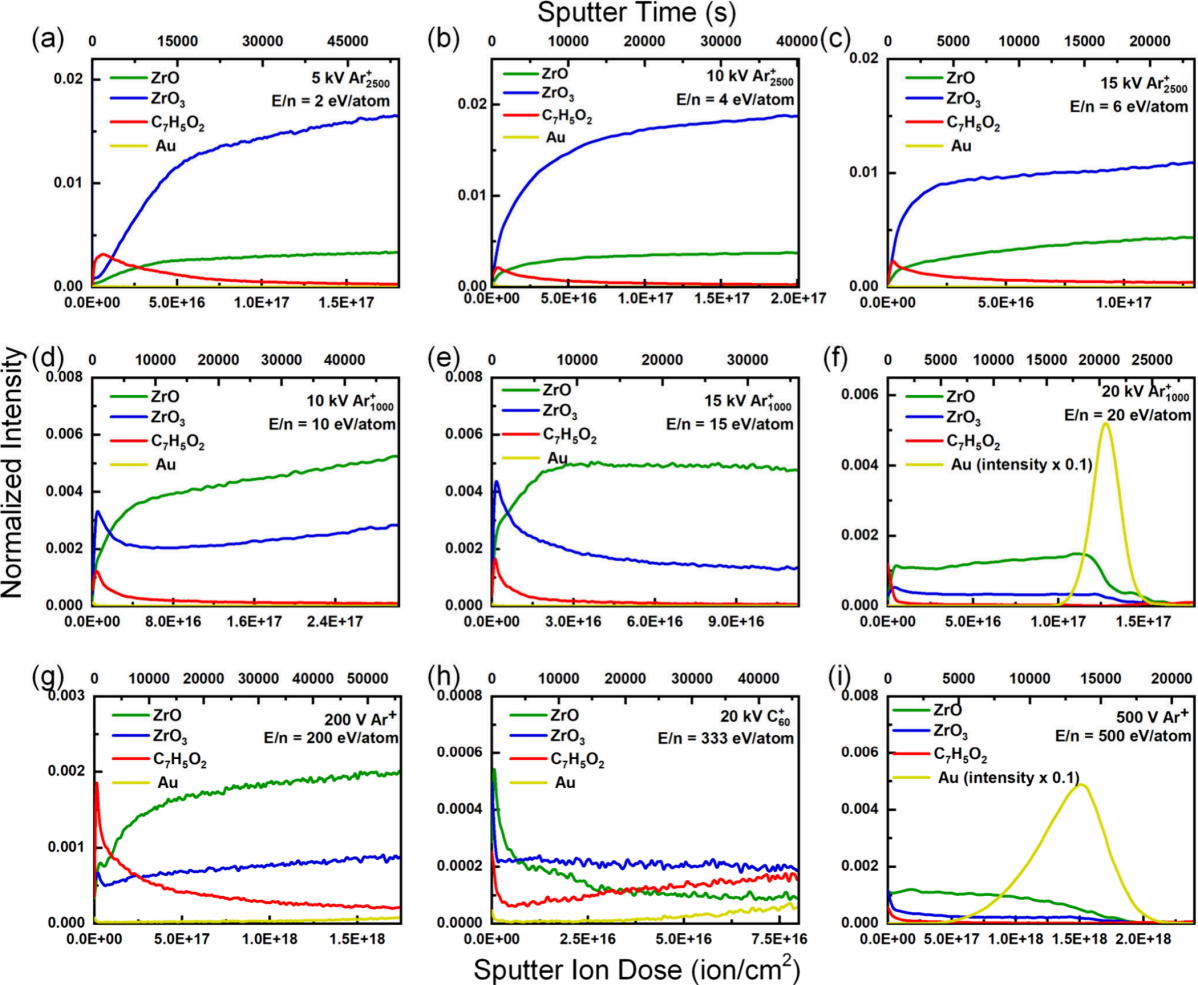
Depth profiles of UiO-66 films obtained with (a) 5 kV Ar_2500_^+^, (b) 10 kV Ar_2500_^+^, (c) 15 kV Ar_2500_^+^, (d) 10 kV Ar_1000_^+^, (e) 15 kV Ar_1000_^+^, (f) 20 kV Ar_1000_^+^, (g) 200 V Ar^+^, (h) 20 kV C_60_^+^, and (i) 500 V Ar^+^.

**Figure 2:**
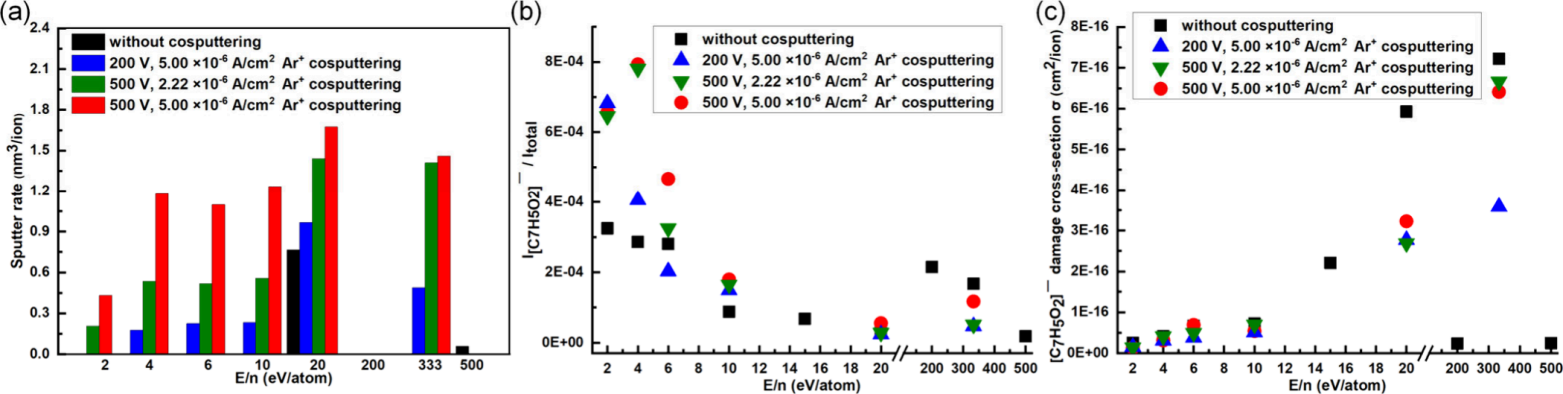
(a) Sputter rate of UiO-66 films, (b) steady-state intensity, and (c) damage cross sections of [C_7_H_5_O_2_]^−^ obtained with different sputter parameters.

In general, the initial increase in representative ions can be attributed to the removal of surface contaminants adsorbed during sample transfer in air. The continuous increase in [ZrO]^−^ and [ZrO_3_]^−^ indicated the fragmentation of the Zr_6_O_4_(OH)_4_ nodes, which yielded smaller clusters on the remaining surfaces. For Ar_2500_^+^ (Figure 1a-1c, E/n = 2–6 eV/atom) sputtering, the intensities of [ZrO_3_]^−^ were greater than those of [ZrO]^−^, indicating that the larger cluster of Ar_2500_^+^ could better preserve the Zr_6_O_4_(OH)_4_ nodes. On the other hand, Ar_1000_^+^ (Figure 1d-1f, E/n = 10–20 eV/atom) and Ar^+^ (Figure 1g and 1i, E/n = 200–500 eV/atom) sputtering tend to fragment the metallic cluster; hence, the intensity of [ZrO_3_]^−^ decreases in the early stage of sputtering (the initial increase is attributed to the removal of surface contaminants). For C_60_^+^ (Figure 1h, E/n = 333 eV/atom) sputtering, [ZrO]^−^ was higher than [ZrO_3_]^−^ was, which also indicated significant fragmentation in the early stage of sputtering. However, the intensity of [ZrO_3_]^−^ decreased less significantly at higher sputter doses. Notably, the intensity of representative ions was lower than that of Ar-based beams because of the significant C deposition, which caused [C_n_]^−^ (*m*/*z* 12 × n) to dominate the spectrum (Figure S4). Therefore, the preservation of metallic nodes after extended sputtering is attributed to graphitic C deposition on the remaining surface, which can dissipate the energy of the ion beam and suppress the fragmentation process.

While a lower E/n (Ar_2500_^+^) could better preserve metallic nodes, it could also preserve the structure of the organic linker and, in turn, preserve the intensity of the organic linker ([C_7_H_5_O_2_]^−^). As a result, when the ion beam induced damage to the chemical structure and removal of this surface damage reached equilibrium, the steady-state intensity was reached. Figure 2b summarizes the intensity of the organic linker at the steady state (numerical values are presented in Table S2). Although Ar_2500_^+^, C_60_^+^ and 200 V Ar^+^ yielded higher intensities, the rapid drop in the early stage of sputtering indicated significant structural damage to the organic linker. To quantify the ion beam-induced chemical damage and removal of damage, an erosion model^52^ was used to fit the (effective) damage cross section (σ) of [C_7_H_5_O_2_]^−^ (Figure 2c; the numerical values are presented in Table S3). With GCIB, a lower E/n led to a lower damage cross-section and better preserved the chemical structure of UiO-66. However, a lower E/n also results in a lower sputter yield, especially for inorganic components, which in turn leads to preferential sputtering of organic components.^29^ As a result, a significant decrease in the intensity of [C_7_H_5_O_2_]^−^ was observed as a joint result of damage to the organic structure and preferential loss of organic components on the remaining surface. Furthermore, especially for lower E/n values, the low sputter rate also prevented successful profiling of the UiO-66 films. For Ar^+^, although low-damage cross sections were observed with acceleration voltages of 200 and 500 V, the low sputter rate and low steady-state intensity, respectively, also prevent their utilization. Owing to the extensive graphitic C deposition and low sputter rate, the high damage cross-section of C_60_^+^ made it undesirable as well. In summary, owing to the high damage cross-section, low sputter rate or low organic ion intensity, commonly used ion beams, including GCIBs, C_60_^+^ and Ar^+^, are not ideal for profiling organic–inorganic hybrid materials such as the UiO-66 MOF presented herein.

### Depth Profile of UiO-66 with Ar^+^ Cosputtering

While GCIBs with a lower E/n could better preserve the chemical structure but could not sputter inorganic components efficiently, cosputter^53–60^ with atomic Ar^+^ that could suppress the preferential sputtering between organic and inorganic components was attempted here. Ideally, the damage introduced by atomic ion beams can be removed by cluster beams; hence, the damage could be masked and molecular information preserved. Earlier work suggested that cosputter with low-energy-high-current atomic beams is suitable for using lower E/n GCIBs and that high-energy-low-current cosputter beams are suitable for higher E/n GCIBs.^53^ Following prior works, 200 V-5.00 × 10^–6^ A/cm^2^ Ar^+^ was used to cosputter with GCIBs and C_60_^+^ (Figure 3; the results are summarized in Figure 2, and the numerical values are presented in Tables S1–S3).

**Figure 3:**
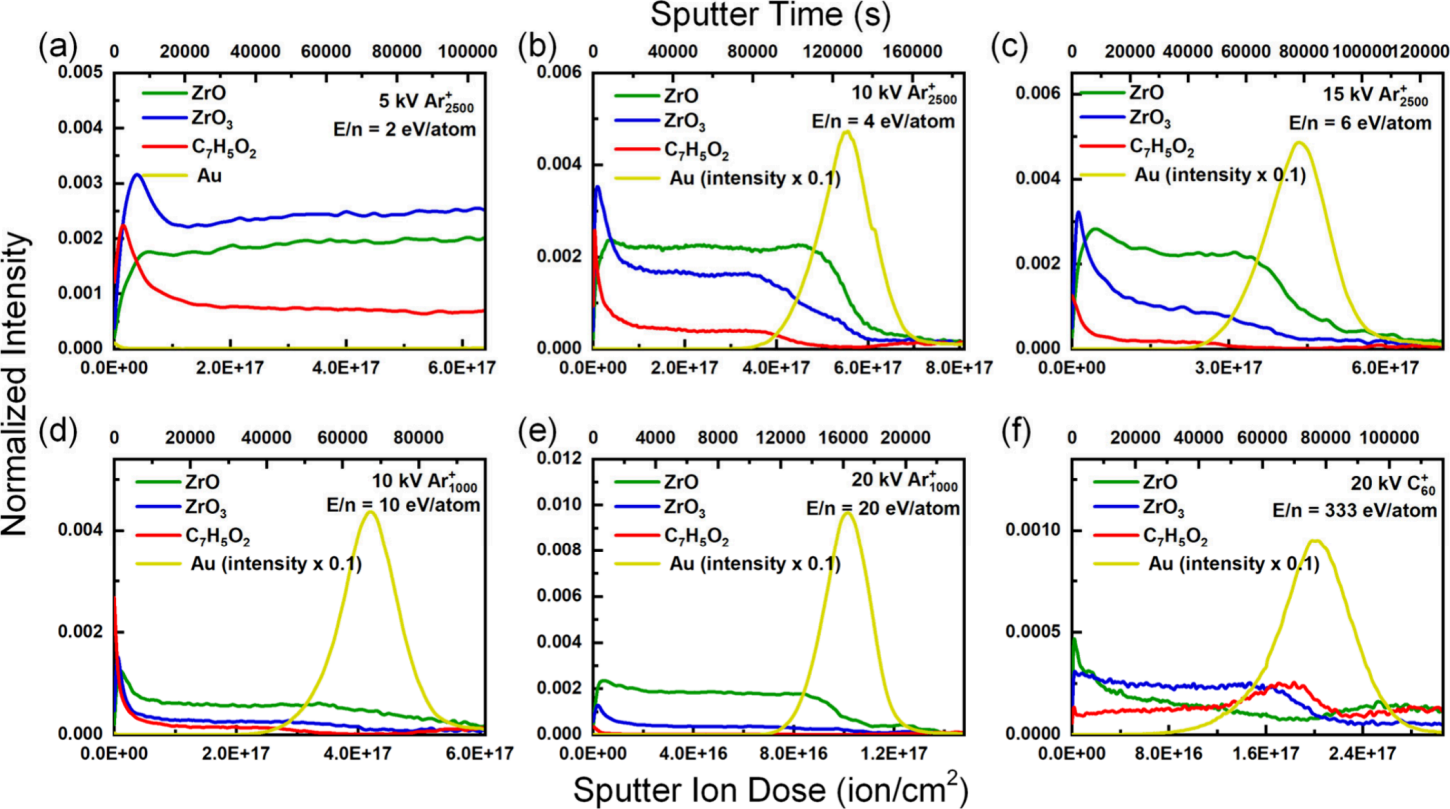
Depth profiles of UiO-66 films produced via 200 V Ar^+^ cosputter with (a) 5 kV Ar_2500_^+^, (b) 10 kV Ar_2500_^+^, (c) 15 kV Ar_2500_^+^, (d) 10 kV Ar_1000_^+^, (e) 20 kV Ar_1000_^+^, and (f) 20 kV C_60_^+^.

With 5 kV Ar_2500_^+^ (E/n = 2 eV/atom, Figure 3a), [ZrO_3_]^−^ and [C_7_H_5_O_2_]^−^ are better preserved, and the steady-state intensity of [C_7_H_5_O_2_]^−^, which represents the organic linker, is greater than that without cosputter (Figure 1a). However, the Au substrate still cannot be exposed in a reasonable amount of time. With increasing E/n (Figure 3b-e), the 200 V Ar^+^ cosputter increased the sputter rate, and the sputter rate increased with increasing E/n (Figure 2a). However, the damage cross-section (Figure 2c) also increased, and a decreasing steady-state intensity (Figure 2b) was observed with increasing E/n. Although a higher sputter rate may suppress preferential sputtering between organic and inorganic components, it is not high enough to sufficiently remove ion-induced damage. For C_60_^+^ (Figure 3f), in addition to the higher sputter rate that allows the exposure of the Au substrate, the general trend is similar to that without cosputter (Figure 1h). Although the damage cross-section was decreased by cosputter (Figure 2c), the steady-state intensity of [C_7_H_5_O_2_]^−^ was also reduced (Figure 2b). In summary, 200 V Ar^+^ cosputter improved the sputter rate and the removal of ion-induced damage, but it did not provide satisfactory results.

Following prior work,^53^ 500 V-2.22 × 10^–6^ A/cm^2^ (high-energy-low-current) Ar^+^ was also employed as the cosputter source (Figure 4). Compared with the 200 V cosputter, the 500 V Ar^+^ cosputter yielded a higher sputter rate (Figure 2a) and similar damage cross sections (Figure 2c) for GCIBs with E/n values of 2–20 eV/atom. Therefore, a high steady-state intensity of [C_7_H_5_O_2_]^−^ can be obtained (Figure 2b). However, for C_60_^+^, which significantly deposits graphitic C, even a high sputter rate can be obtained, the high damage cross-section and low steady-state intensity still produce nonideal results.

**Figure 4:**
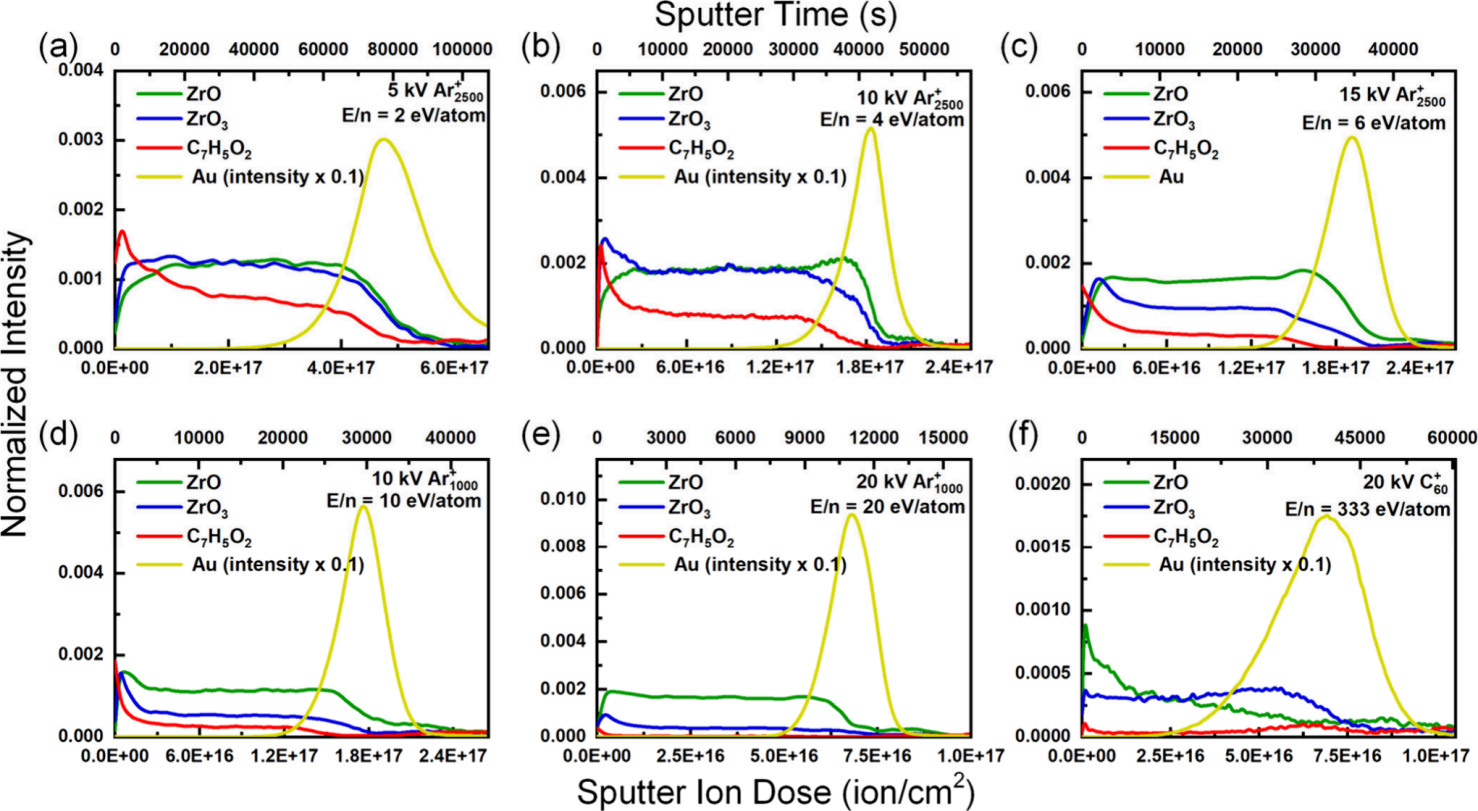
Depth profiles of UiO-66 films produced with 500 V-2.22 × 10^–6^ A/cm^2^ Ar^+^ cosputter with (a) 5 kV Ar_2500_^+^, (b) 10 kV Ar_2500_^+^, (c) 15 kV Ar_2500_^+^, (d) 10 kV Ar_1000_^+^, (e) 20 kV Ar_1000_^+^, and (f) 20 kV C_60_^+^.

To further improve the depth profile, 500 V-5.00 × 10^–6^ A/cm^2^ Ar^+^ cosputter was used (Figure 5). With a higher current, the sputter rate is further increased compared with that of the lower current counterparts (Figure 2a). Furthermore, with a similar damage cross-section (Figure 2b), a higher steady-state intensity can be achieved (Figure 2b). In particular, at E/n = 4 eV/atom (Figure 5b, 10 kV Ar_2500_^+^), the highest [C_7_H_5_O_2_]^−^ was obtained, and a stable intensity of ∼ 50% initial intensity was observed in the UiO-66 layer. In the interface region, a sharp decline in UiO-66-related peaks can also be observed. This ideal depth profile is attributed to the balance between the low ion-induced damage of low E/n GCIB and the higher removal rate of the damaged surface by the Ar^+^ cosputter. Therefore, 10 kV-7.00 × 10^–7^ A/cm^2^ Ar_2500_^+^ with 500 V-5.00 × 10^–6^ A/cm^2^ Ar^+^ cosputter was determined to be the optimized parameter.

**Figure 5:**
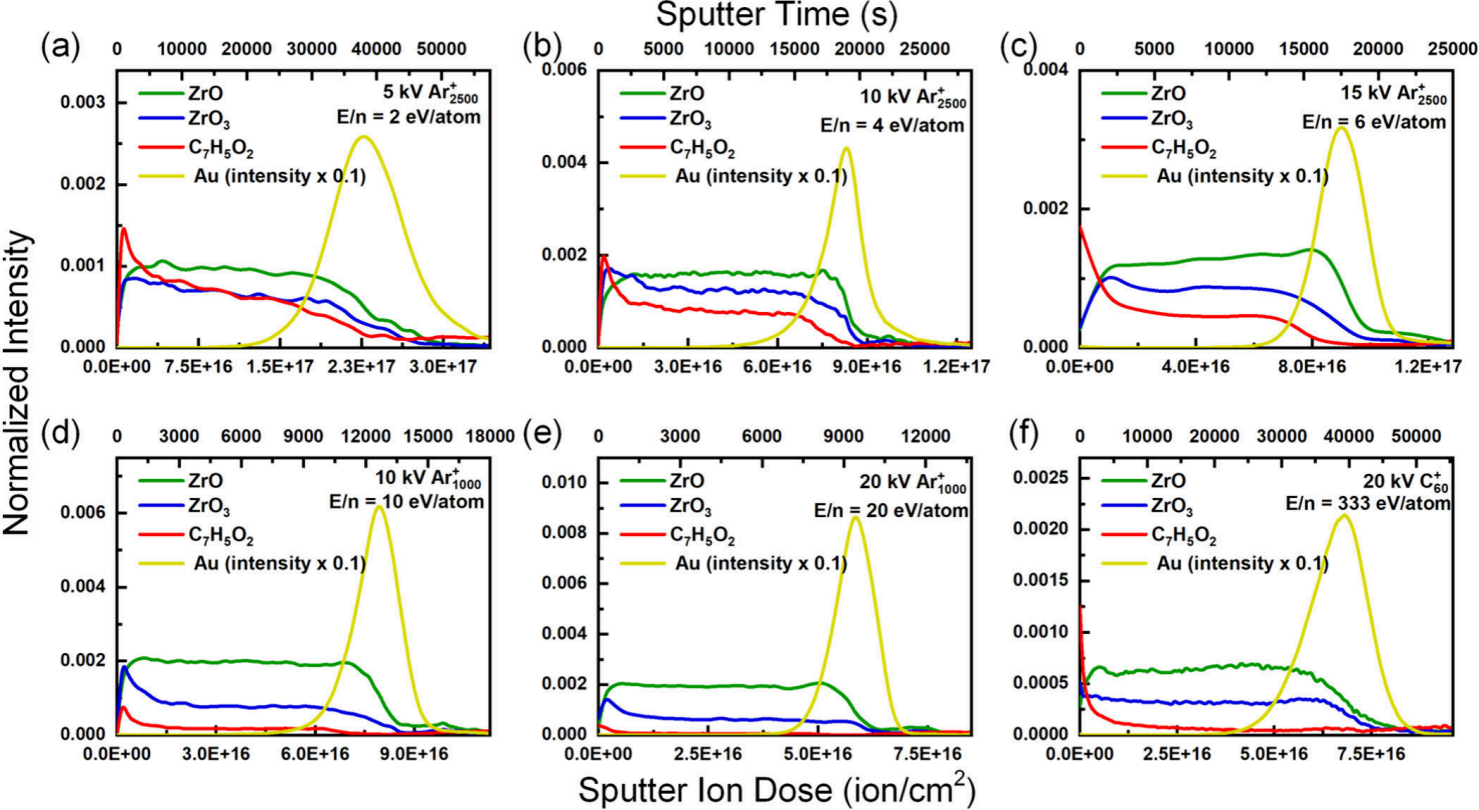
Depth profiles of UiO-66 films produced with 500 V-5.00 × 10^–6^ A/cm^2^ Ar^+^ cosputter with (a) 5 kV Ar_2500_^+^, (b) 10 kV Ar_2500_^+^, (c) 15 kV Ar_2500_^+^, (d) 10 kV Ar_1000_^+^, (e) 20 kV Ar_1000_^+^, and (f) 20 kV C_60_^+^.

### Depth Profile of Caffeine-Loaded UiO-66

To determine if the optimized parameter is suitable for analyzing guest molecules adsorbed inside the cavities of the MOF, caffeine was selected as the modeling guest under the following considerations: 1. It is not ionic and ligand exchange between linker and caffeine would not take place; 2. It does not contain phenyl groups that yield overlapping fragments similar to terephthalic acid in mass spectrometry; 3. It has a longstanding history of being used as a modeling guest^61,62^ and is known to have high (322 mg/g) adsorption capacity in UiO-66.^63^ Figure 6 shows the ToF-SIMS of pure caffeine, empty UiO-66 and caffeine-loaded UiO-66 for selecting nonoverlapping secondary ions that can represent each component. For caffeine (Figure 6a), intense peaks at *m*/*z* 193, 179, 164, 122, 111, 66, and 64, which represent [M-H]^−^, [M-CH_3_]^−^, [M-(CH_3_)_2_]^−^, [C_5_H_2_N_2_O_2_]^−^, [C_4_H_3_N_2_O_2_]^−^, [C_3_NO]^−^ and [C_3_N_2_]^−^, can be identified. Beside of *m*/*z* 122 ([C_5_H_2_N_2_O_2_]^−^), which overlaps with [ZrO_2_]^−^, other peaks show little or no overlap with UiO-66 and can indicate the presence of caffeine. Owing to the difference in the complexity of fragments, they are grouped into high *m*/*z* (193, 179, 164 and 111) and low *m*/*z* (66 and 64) fragments, and the intensities are summed to construct the depth profile. For MOF, although *m*/*z* 121 ([C_7_H_5_O_2_]^−^) and 138 ([ZrO_3_]^−^) may overlap with some fragments of low intensity from caffeine, the significant difference in relative intensities would not significantly interfere with the identification of MOF (Figure 6d). Therefore, *m*/*z* 121 ([C_7_H_5_O_2_]^−^) was selected to represent the organic linker of UiO-66, whereas *m*/*z* 106 ([ZrO]^−^) and 138 ([ZrO_3_]^−^) were selected to represent the inorganic nodes.

**Figure 6:**
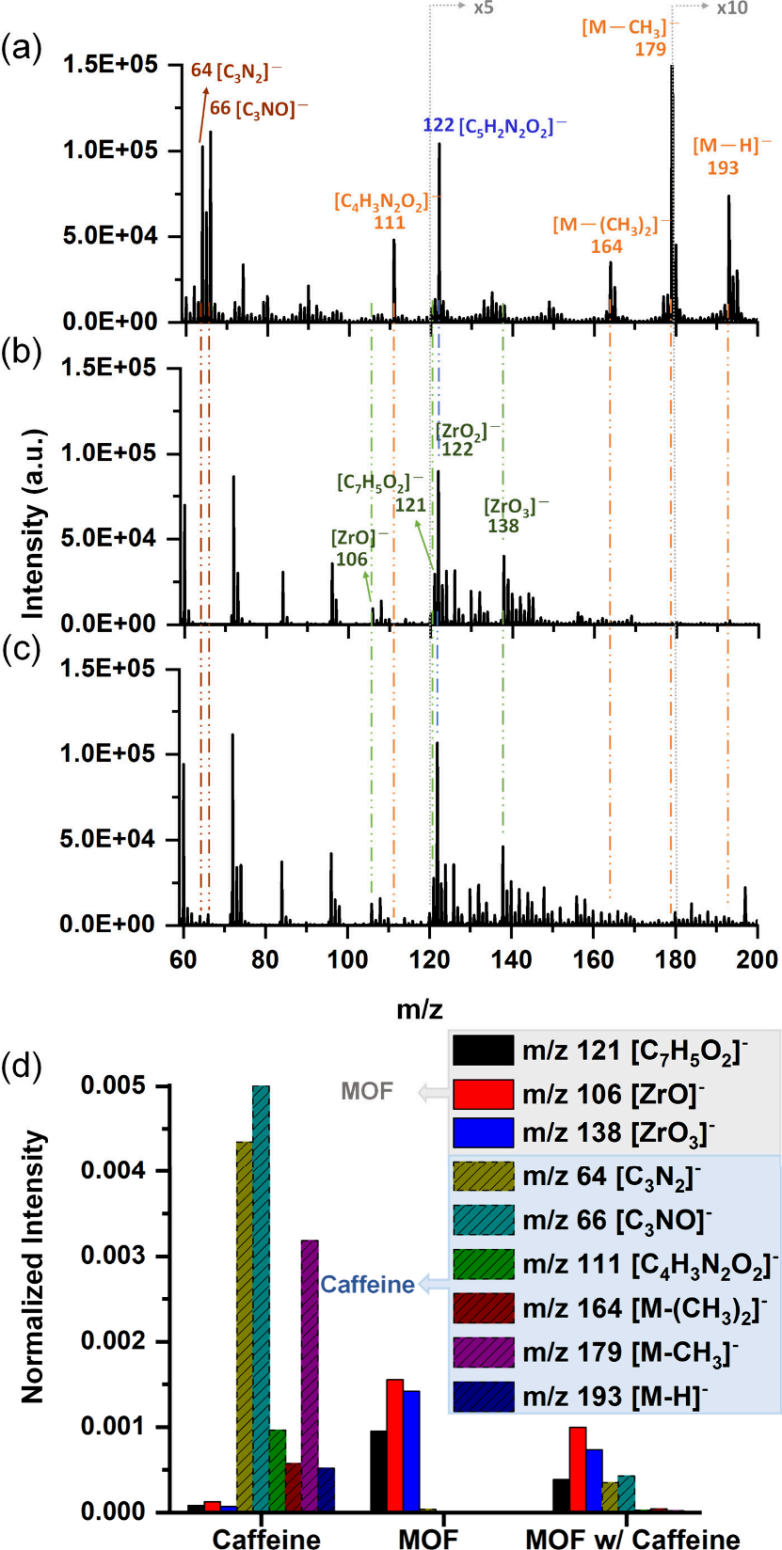
Negative ion ToF-SIMS of (a) pure caffeine, (b) UiO-66, and (c) caffeine-loaded UiO-66. (d) Intensities of selected representative ions normalized with respect to total ion intensity.

Figure 7a shows the reference UiO-66 film on Au obtained with the optimized parameters of 10 kV-7.00 × 10^–7^ A/cm^2^ Ar_2500_^+^ with 500 V-5.00 × 10^–6^ A/cm^2^ Ar^+^ cosputter. As expected, caffeine (CAF)-related ions were not observed within the UiO-66 layer. However, the intensities of the peaks at low *m*/*z* values (64 and 66) appeared to increase inside the Au layer. This artifact is attributed to significant C deposition inside the Au layer^59^ such that the resulting C_5_H_4_ and C_5_H_6_ fragments overlap with the characteristic ions of caffeine. Therefore, ions of low *m*/*z* may not be suitable for determining the spatial distribution of components. Nevertheless, with peaks of higher *m*/*z*, the distribution of caffeine inside UiO-66 could be determined after the loading process (Figure 7b). At the outermost surface (early stage in the depth profile), higher caffeine loading was observed. Considering the similar decreasing trend of [C_7_H_5_O_2_]^−^ from the linker, it is attributed to ion-induced damage. Nevertheless, after this transient region, stable signals from UiO-66 and caffeine were observed, indicating a homogeneous distribution of caffeine inside the bulk of UiO-66. Furthermore, the intensity of caffeine decreased earlier than that of UiO-66, which indicated that the UiO-66/Au interface had a lower caffeine loading. This vertical inhomogeneity can be attributed to the limited diffusion rate of caffeine inside UiO-66, i.e., caffeine cannot reach the bottom interface in a limited time. Furthermore, the decreasing caffeine intensity within the MOF layer indicated that the ion-induced mixing or reorganization of caffeine and UiO-66 might not be a significant factor affecting the depth profile.

**Figure 7:**
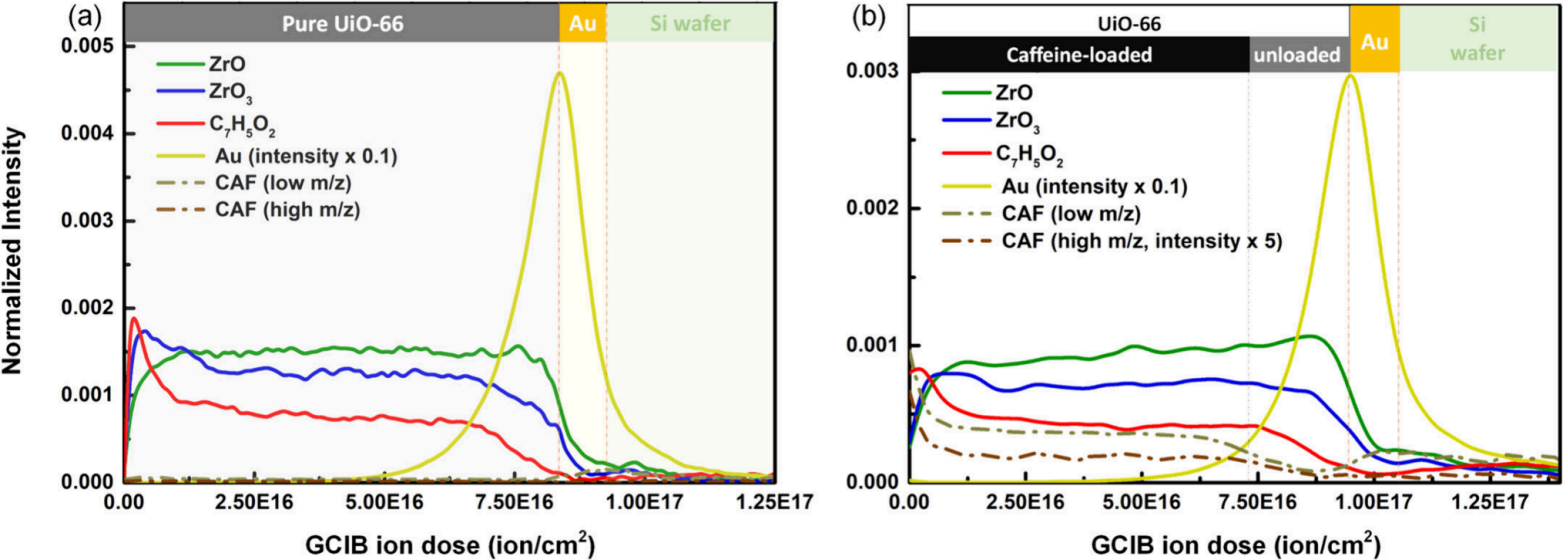
ToF-SIMS depth profile of (a) pure UiO-66 and (b) caffeine-loaded UiO-66 films on a Au substrate obtained with optimized parameters of 10 kV-7.00 × 10^–7^ A/cm^2^ Ar_2500_^+^ with 500 V-5.00 × 10^–6^ A/cm^2^ Ar^+^ cosputter.

The intensity profile of caffeine at the surface was fitted by the solution of Fickian diffusion model^64^ and the diffusivity was found to be 1.35 ± 0.18 × 10^–21^ m^2^/s (R_adj_^2^ = 0.982). In comparison to the reported diffusion coefficient of 2.9 × 10^–19^ m^2^/s for 13.6 wt % caffeine-loaded UiO-66 to release caffeine into distilled water,^65^ the diffusivity of caffeine into the UiO-66 observed in this study is lower. The lower diffusivity observed during the loading process may be attributed to the competition between solvent and caffeine molecules as they are displacing pre-existing molecules within the pores of UiO-66. Conversely, the release process involves a more straightforward exchange between water and caffeine inside the pores of UiO-66.

## Conclusion

In this work, ion beams of energy per atom ranging from 500 V Ar^+^ (E/n = 500 eV/atom) to 5 kV Ar_2500_^+^ (E/n = 2 eV/atom) were used for depth profiling UiO-66 films. Although low damage cross sections can be obtained with low-energy (500 and 200 V) atomic Ar^+^ sputtering, the low sputter rate prevents their application in profiling films. For giant cluster ion beams (GCIBs, Ar_n_^+^, n = 1000, 2500) with higher E/n values, a higher sputter rate could be obtained, which could remove ion-induced damage on the surface more effectively but also cause more damage to the surface; hence, molecular information is lost. With a lower E/n value, although the damage cross-section is lower, the low sputter rate cannot effectively remove ion-induced damage on the surface and cannot sputter through the film. Nevertheless, by using cluster and atomic ion beams at the same time to cosputter the surface, the sputter rate can be increased. With a higher E/n, owing to the more efficient removal of surface damage, the damage cross-section decreases to produce an improved depth profile. With a lower E/n value, the auxiliary Ar^+^ does not introduce additional damage significantly, and the greatly increased sputter rate yields ideal results. With the optimized parameters of 10 kV-7.00 × 10^–7^ A/cm^2^ Ar_2500_^+^ with 500 V-5.00 × 10^–6^ A/cm^2^ Ar^+^ cosputter, the distribution of modeling guest molecules (caffeine) inside the MOF (UiO-66) can be determined.

## Supplementary Material



## Notes

Figure S1, XRD pattern of synthesized UiO-66 and the corresponding reference pattern; Figure S2, topographical AFM and SEM images and cross-sectional images of UiO-66 films spin-coated on Au/Cr-coated Si substrates from particles synthesized with 24, 20.5, and 20 h reaction times; Figure S3, positive and negative ToF-SIMS of UiO-66 and tandem MS data at from positive and negative ToF-SIMS; Figure S4, SIMS of the UiO-66 film after C_60_^+^ sputtering; Table S1, sputter rates of UiO-66 with different sputter parameters; Table S2, steady-state intensity of [C_7_H_5_O_2_]^−^ with different sputter parameters; and Table S3, damage cross sections of [C_7_H_5_O_2_]^−^ with different sputter parameters (PDF)
