# Supplementary material for: Determining the Distributions of Components inside Metal–Organic Framework Thin Films with an Ar-Gas Cluster Ion Beam (Ar1000,2500 +) and Ar+ Cosputter via Secondary Ion Mass Spectrometry
Source: ACS Appl Mater Interfaces. 2025 Jun 17;17(26):38658–68. doi: 10.1021/acsami.5c05778 (PMC12232284; doi:10.1021/acsami.5c05778)
Supplement: Supplementary file 1 [file am5c05778_si_001.pdf]

Supporting Information

Determining the Distributions of Components inside Metal–Organic Framework Thin Films with an Ar-Gas Cluster Ion Beam ($Ar_{1000,2500}^+$) and Ar^+ Cosputter via Secondary Ion Mass Spectrometry

Peng-Hsuan Chiang,[1,2,§] Pochun Hsieh,[1,2§] Cheng-Hung Hou,[1] Yun-Wen You,[1] Man-Ying Wang,[1,3] Ting-Jia Yang[1,3] and Jing-Jong Shyue*[1,2,3]

[1] Research *Center for Applied Sciences, Academia Sinica, Taipei 11529, Taiwan*

[2] Department *of Materials Science and Engineering, National Taiwan University, Taipei 10617, Taiwan*

[3] Program *in Semiconductor Devices, Materials, and Hetero-integration, Graduate School of Advanced Technology, National Taiwan University, Taipei 10617, Taiwan*

[§] Equal contribution

*To whom correspondence should be addressed. Telephone +886(2)2787-3137. Fax +886 (2)2787-3122. E-mail: shyue@gate.sinica.edu.tw

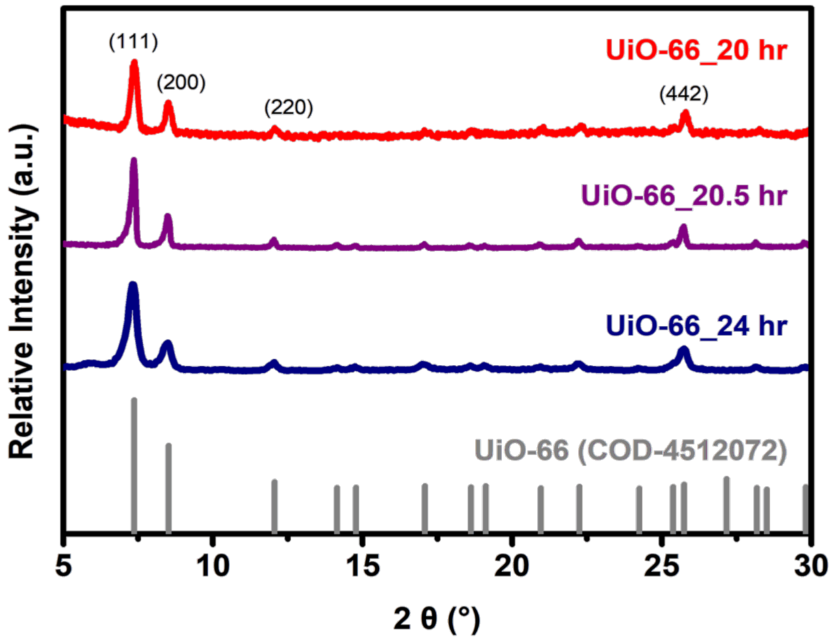

Figure S1. XRD pattern of synthesized UiO-66 and the corresponding reference pattern.

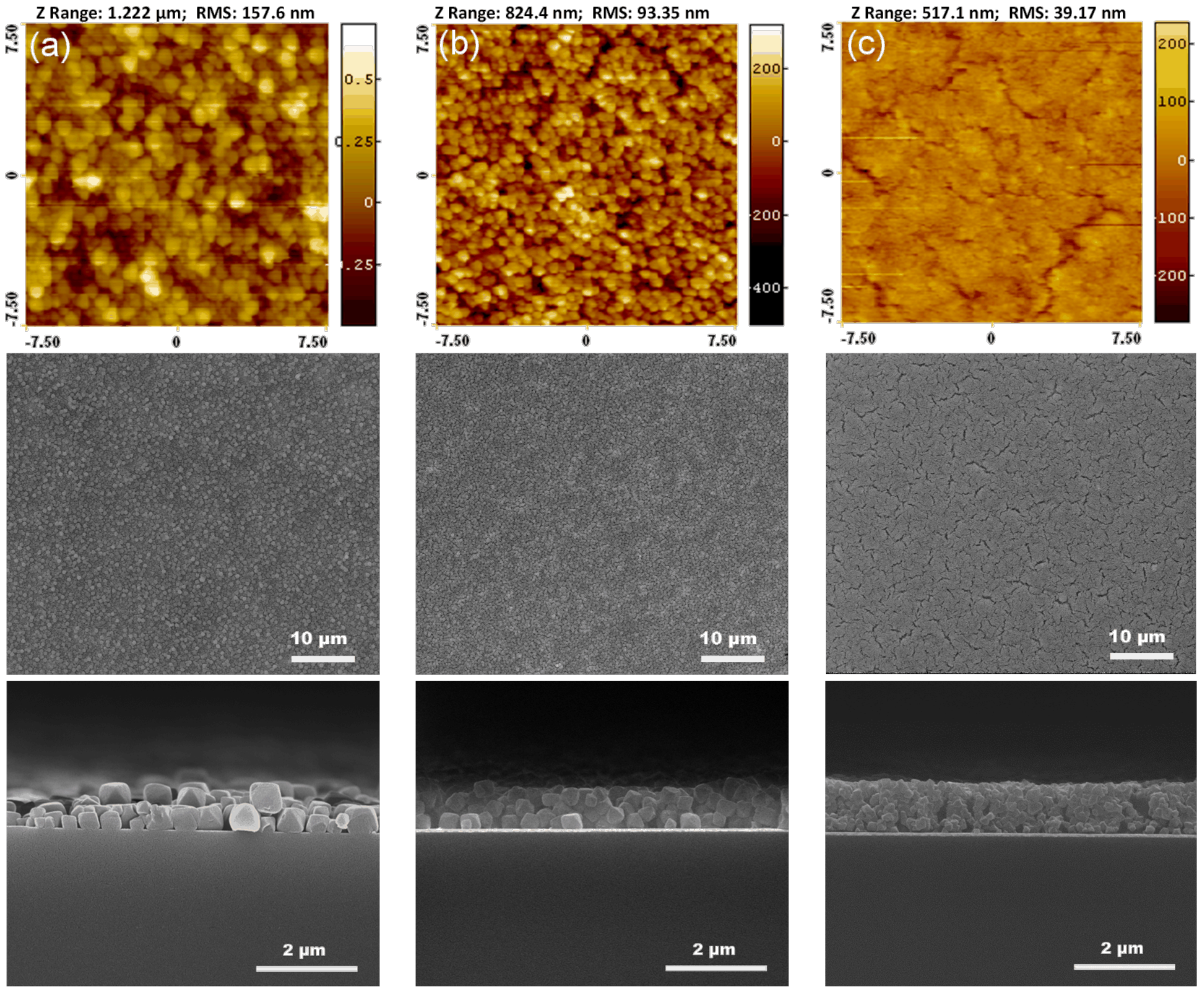

Figure S2. Topographical AFM and SEM images and cross-sectional images of UiO-66 films spin-coated on Au/Cr-coated Si substrates from particles synthesized with **(a)** 24, **(b)** 20.5 and **(c)** 20 h reaction times. The field-of-view of the AFM images is 15×15 μm^2.

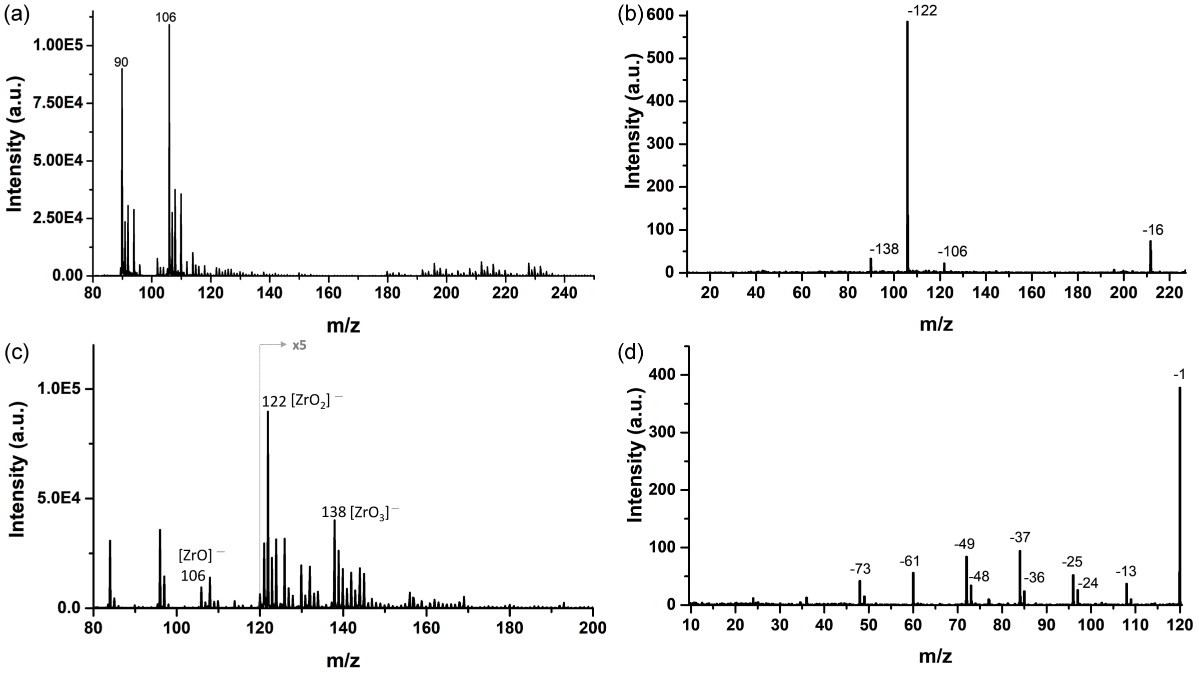

Figure S3. (a) Positive and **(c)** negative ToF-SIMS of UiO-66, **(b)** and **(d)** tandem MS data at m/z 228 and 121 from positive and negative ToF-SIMS, respectively.

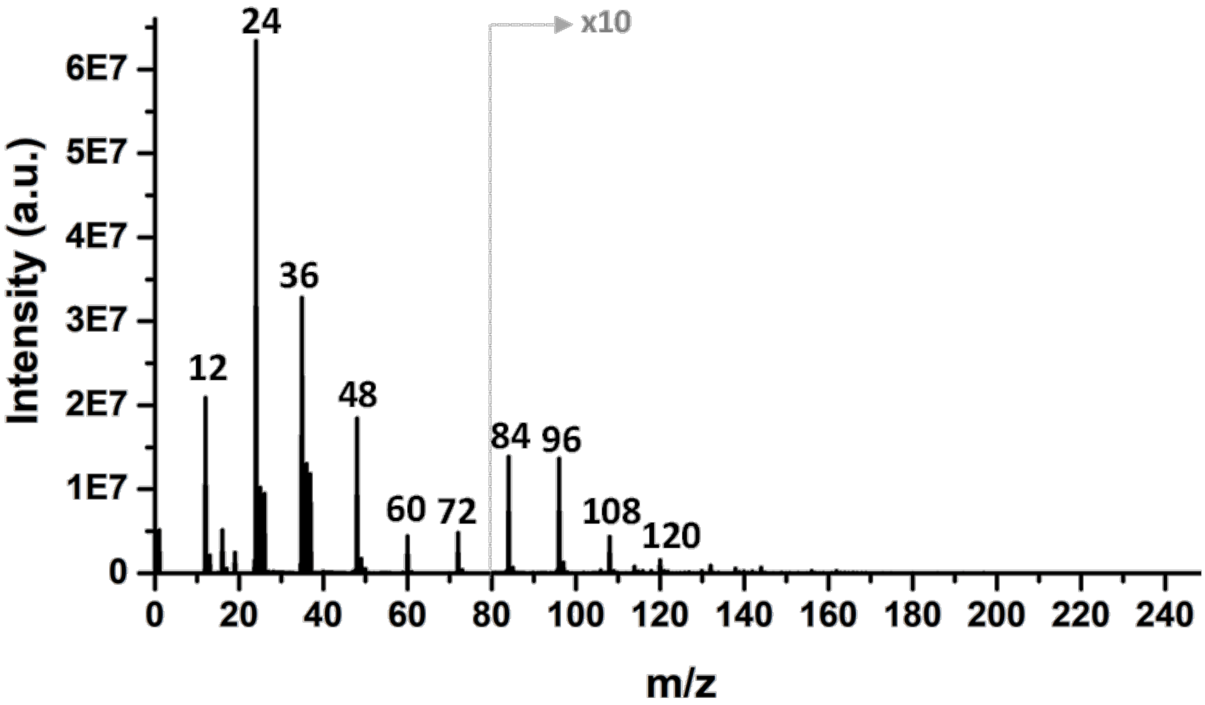

Figure S4. SIMS of the UiO-66 film after C_{60}^{+} sputtering.

Table S1. Sputter rates of UiO-66 with different sputter parameters.

Sputter parameter		without cosputtering	200 V, 5.00×10^{-6} A/cm^2 Ar^+ cosputtering	500 V, 2.22×10^{-6} A/cm^2 Ar^+ cosputtering	500 V, 5.00×10^{-6} A/cm^2 Ar^+ cosputtering
		Sputter rate (nm^3/ion)			
500 V Ar^+		0.06	N/A	N/A	N/A
200 V Ar^+		-	N/A	N/A	N/A
20 kV C_{60}^+		-	0.49	1.41	1.46
GCIB, Ar_n^+	E/n = 2	-	-	0.21	0.43
	E/n = 4	-	0.18	0.54	1.18
	E/n = 6	-	0.28	0.52	1.10
	E/n = 10	-	0.23	0.56	1.23
	E/n = 15	-	N/A	N/A	N/A
	E/n = 20	0.77	0.97	1.44	1.67

Table S2. Steady-state intensity of $[C_7H_5O_2]^-$ with different sputter parameters.

Sputter parameter		without cosputtering	200 V, 5.00×10^{-6} A/cm^2 Ar$^+$ cosputtering	500 V, 2.22×10^{-6} A/cm^2 Ar$^+$ cosputtering	500 V, 5.00×10^{-6} A/cm^2 Ar$^+$ cosputtering
		$I_{[C7H5O2]^-}$ / I_{total}, ($\times10^{-4}$)			
500 V Ar$^+$		0.18±0.05	N/A	N/A	N/A
200 V Ar$^+$		2.15±0.16	N/A	N/A	N/A
20 kV C_{60}^+		1.67±0.10	1.17±0.06	0.47±0.07	0.51±0.07
GCIB, Ar_n^+	E/n = 2	3.25±0.13	6.83±0.94	6.45±0.28	6.57±0.51
	E/n = 4	2.86±0.21	4.05±0.16	7.81±0.29	7.93±0.28
	E/n = 6	2.80±0.17	2.03±0.22	3.25±0.22	4.67±0.22
	E/n = 10	0.88±0.07	1.49±0.07	1.65±0.06	1.79±0.07
	E/n = 15	0.68±0.03	N/A	N/A	N/A
	E/n = 20	0.36±0.05	0.24±0.02	0.28±0.03	0.55±0.11

Table S3. Damage cross-sections of $[C_7H_5O_2]^-$ with different sputter parameters.

Sputter parameter		without cosputtering	200 V, 5.00×10^{-6} A/cm^2 Ar^+ cosputtering	500 V, 2.22×10^{-6} A/cm^2 Ar^+ cosputtering	500 V, 5.00×10^{-6} A/cm^2 Ar^+ cosputtering
		$[C_7H_5O_2]^-$ damage cross-section σ (10^{-17} cm^2/ion)			
500 V Ar^+		2.49*	N/A	N/A	N/A
200 V Ar^+		2.37*	N/A	N/A	N/A
20 kV C_{60}^+		72.2±1.6, R^2_{adj}=0.907	35.8*	66.6*	64.1±1.0, R^2_{adj}=0.924
GCIB, Ar_n^+	E/n = 2	2.55±0.06, R^2_{adj}=0.987	1.37±0.14, R^2_{adj}=0.951	1.55±0.59, R^2_{adj}=0.745	1.45±0.19, R^2_{adj}=0.853
	E/n = 4	4.15±0.30, R^2_{adj}=0.974	3.15±0.30, R^2_{adj}=0.888	3.11±0.25, R^2_{adj}=0.848	4.13±0.50, R^2_{adj}=0.787
	E/n = 6	6.71±0.12, R^2_{adj}=0.857	3.86±0.66, R^2_{adj}=0.943	4.93±0.39, R^2_{adj}=0.987	6.91±0.24, R^2_{adj}=0.782
	E/n = 10	7.24±0.47, R^2_{adj}=0.988	5.41±0.74, R^2_{adj}=0.881	5.12±0.51, R^2_{adj}=0.926	7.00±0.90, R^2_{adj}=0.822
	E/n = 15	22.1±2.3, R^2_{adj}=0.944	N/A	N/A	N/A
	E/n = 20	59.3*	32.3±5.0, R^2_{adj}=0.912	27.7±6.0, R^2_{adj}=0.836	26.9±5.1, R^2_{adj}=0.900

*The raw intensity decreased to a steady state abruptly and there are not enough data points in the transient region to calculate a meaningful standard error.
